# Stress, Inflammation, and Cellular Vulnerability during Early Stages of Affective Disorders: Biomarker Strategies and Opportunities for Prevention and Intervention

**DOI:** 10.3389/fpsyt.2014.00034

**Published:** 2014-04-09

**Authors:** Adam J. Walker, Yesul Kim, J. Blair Price, Rajas P. Kale, Jane A. McGillivray, Michael Berk, Susannah J. Tye

**Affiliations:** ^1^Department of Psychiatry and Psychology, Mayo Clinic, Rochester, MN, USA; ^2^School of Psychology, Deakin University, Melbourne, VIC, Australia; ^3^School of Engineering, Deakin University, Geelong, VIC, Australia; ^4^School of Medicine, Deakin University, Geelong, VIC, Australia; ^5^Department of Psychiatry, University of Melbourne, Melbourne, VIC, Australia; ^6^Orygen Youth Health Research Centre, Melbourne, VIC, Australia; ^7^The Florey Institute of Neuroscience and Mental Health, Melbourne, VIC, Australia; ^8^Department of Psychiatry, University of Minnesota, Minneapolis, MN, USA

**Keywords:** prodrome, depression, bipolar, biomarker, stress, inflammation, cellular resilience, plasticity

## Abstract

The mood disorder prodrome is conceptualized as a symptomatic, but not yet clinically diagnosable stage of an affective disorder. Although a growing area, more focused research is needed in the pediatric population to better characterize psychopathological symptoms and biological markers that can reliably identify this very early stage in the evolution of mood disorder pathology. Such information will facilitate early prevention and intervention, which has the potential to affect a person’s disease course. This review focuses on the prodromal characteristics, risk factors, and neurobiological mechanisms of mood disorders. In particular, we consider the influence of early-life stress, inflammation, and allostatic load in mediating neural mechanisms of neuroprogression. These inherently modifiable factors have known neuroadaptive and neurodegenerative implications, and consequently may provide useful biomarker targets. Identification of these factors early in the course of the disease will accordingly allow for the introduction of early interventions which augment an individual’s capacity for psychological resilience through maintenance of synaptic integrity and cellular resilience. A targeted and complementary approach to boosting both psychological and physiological resilience simultaneously during the prodromal stage of mood disorder pathology has the greatest promise for optimizing the neurodevelopmental potential of those individuals at risk of disabling mood disorders.

## Introduction

There is increasing appreciation for the need to both identify and treat mood disorders during their earliest stages (1). Although some dispute remains, maladaptive changes in mood and behavior first become evident during the prodromal period (2). However, the low specificity of these changes makes the prodromal stage difficult to definitively characterize prior to disease onset (3). Observable changes in mood and general physiologic functioning can include increases in sadness, anhedonia, irritability, anger, and anxiety, together with alterations in sleep and energy (4). Correlating these symptoms with prodromal biomarkers offers an exciting juncture whereby targeted interventions could be opportunistically employed to prevent neurodegenerative changes from accruing as the disease progresses (5). The potential to intervene during the prodromal stage of psychiatric illness through the detection and remediation of novel biomarkers has perhaps been best studied in schizophrenia, wherein most individuals experience a lengthy prodromal period prior to the full emergence of diagnosable psychotic symptoms (6). As an exemplar, low levels of nervonic acid appear to be a risk factor for conversion from high-risk to frank psychosis (7), and this risk of conversion may be reduced by targeted omega-3 fatty acid supplementation (8). Encouraging results from this work have renewed interest in the early detection of affective disorders, particularly bipolar disorder, with the hope that earlier and more targeted interventions might slow disease progression (3, 9–12). This can significantly impact neuroprogression and subsequent disease course for the individual (13). This concept of “neuroprogression” refers to the cumulative restructuring of the central nervous system which in turn mediates the development and persistence of psychiatric illness (14, 15). This process results from disturbances in inflammatory mediators, neurotrophins, oxidative stress, and energy regulation (14, 15).

## Biomarker Strategies for Prodromal Mood Disorders

### Stress and allostatic load

#### Stress sensitization and early detection

Stress is one of the best-studied mediators by which genetic vulnerabilities are translated into mood disorder pathology through the process of neuroprogression (16–18). Numerous studies have demonstrated that both depression and bipolar disorder are more prevalent in individuals who have experienced adverse early-life events. This is partly because such experiences prime future physiologic and neural responses to stress, elicit a state of chronic inflammation (19), alter cellular mediators of plasticity and energy metabolism, and increase cellular “wear and tear” (20–22). Early-life stress (2) can be particularly deleterious because of its potential to influence the programing of the hypothalamic–pituitary–adrenal (HPA) axis (23) to induce persistent sensitization of neuroendocrine, autonomic, oxidative, and immune responses to stress. Over time these sensitized systems cumulatively contribute to the cellular and synaptic alterations underlying neuroprogression (21, 24–26). Specific examples include changes in reactivity of inflammatory cytokines [e.g., interleukin 6 (IL-6)] (25), alterations in markers for lipid peroxidation [e.g., 8-iso-prostaglandin F (2α)], oxidative damage to DNA (8-hydroxy-2′-deoxyguanosine) and RNA (8-hydroxyguanosine) (24), as well as altered cortisol, adrenocorticotropic hormone, and corticotrophin releasing factor responses (26). Identification of the state of physiologic and cellular resilience or sensitivity to stress may provide an important indicator of the level of neuroprogression and stress-mediated disease pathology for affective disorders, potentially prior to the initial manifestation of the mood episode (22).

One mechanism whereby HPA axis sensitization is likely to occur is through epigenetic regulation of stress response processes (21, 27). Evidence shows that exposure to various forms of stress result in multiple epigenetic changes in limbic regions as well as the HPA axis (21, 27). Interestingly, a recent study by Klendel and colleagues (18) found that only individuals who exhibited allele-specific DNA demethylation in functional glucocorticoid response elements of FK506 binding protein 5 (*FKBP5*), were prone to developing persistent cortisol dysregulation (18, 21). Further, this association was found to be dependent on an interaction effect with trauma in early life, suggesting that key developmental stages are directly related to stability of the observed effects across time (18). In another study, significant interactions between peripheral *FKBP5* mRNA expression and disease progression were reported, suggesting that polymorphisms in the gene directly impact the extent of neuroendocrine dysregulation, and corresponding neuroprogression (28). The *FKBP5* risk allele and corresponding levels of mRNA expression may represent useful biomarkers. These markers could be employed to identify individuals in the prodromal stages of stress-sensitive psychiatric disorders, such as major depression or bipolar disorder. Such detection would facilitate early intervention and could improve resilience and alleviate allostatic load in the prodromal individual.

#### Early-life stress and accumulation of allostatic load

Accumulation of allostatic load is a key mechanism through which early-life stress is thought to result in psychopathology (29). This is mediated via a series of enduring adaptive changes across a range of systems primed both to respond rapidly to challenge, as well as to restore homeostatic equilibrium (30). Adaptive allostatic mechanisms may fail when chronically challenged or when regulatory systems falter. This leads to a state of allostatic overload, which is thought to considerably impact the clinical course of mood disorders (31–33). Without sufficient opportunity for recovery, the brain and body are repeatedly exposed to molecular mediators of stress that can increase the level of cellular “wear and tear” (33). These mediators, which include metabolic factors, inflammatory cytokines, neurotrophins, and oxidative species, collectively impact an individual’s mental and physical resilience as outlined below [for more detailed reviews see Ref. (6, 34, 35)]. Both physiological (i.e., immune and/or metabolic) and psychological (i.e., bullying) stressors contribute significantly to allostatic load, and thus need to be considered together when assessing both risk and relative staging of mood disorder pathology (6, 34).

Enhancing an individual’s capacity to buffer the physiologic toll that accumulates through allostatic overload should be considered an important early intervention strategy. As allostatic load accumulates and attempts to maintain cellular homeostasis fail, cell danger signals are propagated and pro-apoptotic cell signaling pathways become increasingly engaged (36–39). This may play a role in medical comorbidities such as heart disease (40), as well as interfere with the therapeutic mechanisms of antidepressants and mood stabilizers to impair treatment efficacy (41–43). Internal stressors that activate the HPA axis and associated allostatic systems can limit an individual’s capacity for allostasis even prior to the onset of external stressors (36). For example, an endogenous load can build through the expression of homocysteine or inflammatory cytokines, limiting the capacity of adaptive responses in the face of subsequent stressors. Interventions that counter this load and reduce levels of proinflammatory mediators or interfere with their neuromodulatory actions could limit neuroprogression in both bipolar and unipolar depression, as well as enhance capacity for antidepressant efficacy (44–46).

### Inflammatory profile

Stress during earlier life is not only associated with disruption of the HPA axis, but may also serve to sensitize proinflammatory responses to future insults (47–49). Inflammatory mechanisms are increasingly appreciated for their critical role in mood disorder pathophysiology, in particular via their regulation of neuronal excitability, synaptic transmission, synaptic plasticity and neuronal survival (41, 50, 51). Of specific interest are proinflammatory mediators, such as cytokines [i.e., interleukin 1, IL-6, and tumor necrosis factor alpha (TNF-α)] and C-reactive protein (CRP). CRP is often used as a biomarker for inflammation in studies due to its relationship with proinflammatory cytokines and role in the immune response. As demonstrated by Slopen and colleagues (49), individuals at ages 10 and 15 who reported adverse life events at critical stages between the ages of 1.5 and 8 years were found to have significantly increased levels of CRP and IL-6. These heightened concentrations were correlated with immune activation and depressive-like symptoms. Notably, increased CRP levels have been used previously to predict depression severity and recurrence rates in males (48, 52).

There is a growing literature supporting the use of inflammatory biomarkers as predictors of ensuing mood disorder pathology (22). Research to date has been focused on investigating the relationship between inflammatory cytokines and affective disorders in adults; however, their specific role in early onset/adolescent psychopathology is less well explored (53). Cytokines are thought to influence neurodevelopment during key stages, such as adolescence, interacting with biological systems including those of stress hormones and gonadal hormones (53). As such, perturbation of inflammatory balance in adolescents may significantly contribute to neuroprogression and development of psychiatric illness (19, 53, 54). For example, elevated serum levels of TNF-α, IL-6, and interleukin-10 (IL-10) have been reported during the early stages of bipolar disorder (55), and CRP appears to be a biomarker of *de novo* depression risk (56).

As the mood disorder pathology progresses, an increasing number of proinflammatory cytokines are observed, including elevated levels of interferon gamma (IFN-γ) (22, 54, 55). Notably, increases in IFN-γ are associated with dysregulation of the tryptophan metabolite pathway via direct role in indoleamine 2,3-dioxygenase (IDO) activation. Activation of IDO is commonly found in later stages of mood disorders, and is a biomarker of depression-like behavior mediated by neural inflammation in animal models (48). Proinflammatory cytokines activate IDO, resulting in depletion of serotonin and augmentation of quinolinic acid (QUIN) metabolism over kynurenic acid (KYNA). Tryptophan metabolites (kynurenine, KYNA, 3-hydroxykynurenine, and QUIN) act as neuromodulators to influence behavioral, neuroendocrine, and neurochemical aspects of depression (57–60). Consequently, this accumulation of QUIN facilitates neurodegeneration over neuroprotection, impacting mood disorder neuroprogression and resultant disability (61).

It is noteworthy to mention several other findings regarding altered inflammation in youth with psychiatric pathology. Increased mRNA and protein expression levels of IL-1β, IL-6, and TNF-α were reported in the anterior prefrontal cortex of adolescent suicide victims compared with normal control subjects (62). Elevated levels of inflammatory cytokines (among others: TNF-α, IL-1β, IL-6, and IFN-γ) were also observed in the serum of pediatric patients who experienced first-episode psychosis, in addition to increased leukocyte counts and evidence of blood–brain barrier damage (63). Quantification of inflammatory biomarkers (e.g., TNF-α, IL-6, IL-10, or CRP) may thus prove useful for detecting individuals at risk for developing a mood disorder. A recent study by Byrne and colleagues (64) suggests that levels of peripheral cytokines (e.g., IFN-γ) and CRP in salivary samples may correlate with serum samples in young people. Salivary assay may prove to be a simpler, less invasive method of estimating peripheral levels of inflammatory markers in adolescents (64). This provides one avenue whereby prodromal individuals could potentially be identified and their disease onset delayed.

### Diminished synaptic integrity

Homeostatic control of synaptic connections within key mood-related circuits plays a critical role in the etiology of mood disorders (65). Stress and inflammation as discussed in previous sections are implicated in disruption of synaptic signaling and integrity during the early stages of mood disorder pathogenesis. This is mediated in part through the inhibition of neurotrophin function, of which brain derived neurotrophic factor (BDNF) is the most thoroughly characterized. BDNF plays an important role in neuronal development, survival, and function, including activity-dependent synaptic plasticity (66). Synaptic plasticity is characterized by various processes, including synaptic remodeling, synaptogenesis, long-term potentiation, and long-term depression, all of which critically mediate the flow of electrochemical information throughout the central nervous system (67, 68). Stress, allostatic load, inflammation, antidepressants, and mood stabilizers exert major effects on signaling pathways that regulate cellular plasticity, suggesting these are critical neurobiological mediators of mood dysfunction and therapeutic intervention (69–72).

Glycogen synthase kinase-3 (GSK-3), part of the signaling cascade regulated by BDNF, plays an important role in synaptic homeostasis through regulation of synaptic deconsolidation (pruning) and glutamate receptor cycling (73). Increased GSK-3-mediated synaptic deconsolidation has been suggested to be an important factor contributing to reduced spine density in mood disorders (74). Additionally, levels of activated GSK-3 are increased in postmortem brain tissue from individuals with unipolar and bipolar depression (74). In addition to BDNF, GSK-3 is deactivated by signals originating from numerous signaling pathways demonstrated to be dysregulated in mood disorders (e.g., Wnt and PI3K pathways), and is either the direct or downstream target of many mood stabilizer and antidepressant medications (75). GSK-3 activity is modulated by serotonin and dopamine, and is a critical node at the intersection of multiple neurotransmitter and cell signaling cascades (68). As a result, GSK-3 modulates not only synaptic plasticity but also apoptotic mechanisms and, in turn, plays a critical role in mediating cellular resilience (75). For this reason, GSK-3 has received much attention for its potential to be targeted as an early intervention strategy during the prodrome period.

### Identifying impaired cellular resilience

Stress, allostatic overload, and neuroinflammation function together to impair synaptic plasticity and cellular resilience. Disrupted plasticity along with increased cellular vulnerability contributes significantly to the pathophysiology of mood disorders and directly to the neuroprogressive nature of the disease course (3, 76). Some of the key mechanisms of disease progression affecting cellular resilience include: oxidative stress, decreased neurotrophic factor expression, reduced neurogenesis, impaired regulation of calcium, altered endoplasmic reticulum and mitochondrial function, together with dysregulated energy metabolism and insulin signaling. Each of these mechanisms are mediated by allostatic overload and neuroinflammation [for detailed reviews see Ref. (3, 36, 76–78)]. Together, these processes demonstrate that in addition to synaptic integrity, maintenance of cellular homeostasis is critical for facilitating cellular resilience and attenuating mood disorder pathogenesis (79), which is also likely to enhance the capacity for treatment response during later stages of the disorder (80).

Cellular vulnerability and resilience are mediated by apoptotic and anti-apoptotic intracellular signaling cascades, respectively. Apoptosis is important for the regulation of developmental processes and prevention of cancerous growths. Excessive apoptosis in neuronal systems, however, leads to neurodegeneration and certain cell populations are at increased risk of stress-mediated apoptotic cell death (80). Apoptosis is a tightly regulated and energy-dependent process, which coordinates programed cell death in response to different stimuli (81). This can occur through stimulation of death receptor proteins [i.e., tumor necrosis factor (TNF) receptor] by cytokines of the TNF superfamily or in response to mitochondrial degradation. These stimuli result in activation of executioner caspases that function to coordinate cellular process necessary for apoptosis, including cessation of cell repair processes and cell cycle progression, cytoskeletal and nuclear disassembly, and flagging the cell for phagocytosis (82). Distinct classes of antidepressants and mood stabilizers have been demonstrated to facilitate cellular resilience to prevent progression of pro-apoptotic processes, and novel treatments are currently being developed to target these specific mechanisms (83). Biomarkers that characterize the level of neuronal vulnerability relative to resilience may prove useful as biomarkers of prodromal mood disorder pathology. This has been demonstrated for later stages of bipolar disorder (84), however more studies are needed to determine the utility of such cell danger biomarkers during the mood disorder prodrome (22).

## Opportunities for Prevention and Intervention

### Identifying vulnerabilities and building resilience at the cellular level

**Figure 1:**
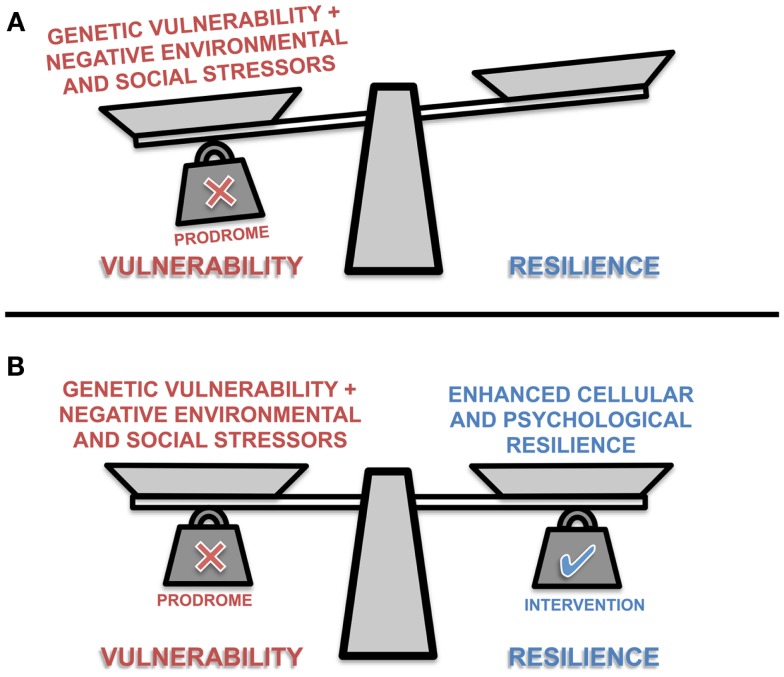
**A representation of the conceptual balance between vulnerability and resilience in prodromal individuals**. The scale’s balance beam teeters between vulnerability and resilience as scale pans are loaded with different positive and negative biological, psychological and social factors. The presence or absence of these factors influence the ability of the individual to cope with stressors, and maintain allostasis. **(A)** Prodromal individuals are somewhat predisposed to vulnerability; but with intervention **(B)** an individual may adopt more adaptive environmental coping strategies, support mechanisms, general healthy lifestyle choices, and/or receive pharmacological interventions that collectively enhance physiological and psychological resilience.

Identification of individuals at risk of developing a mood disorder, or those in the prodromal stage, provides a potential opportunity to target these mechanisms for neuroprotective interventions that enhance cellular resilience, maintain synaptic plasticity and boost psychological resilience (Figure 1) (85). One of the longest held notions of brain plasticity is that certain critical periods or windows exist in development, during which circuitry is consolidated for lifetime functionality. Recently, there is a rising consensus that developmentally induced plasticity can, to an extent, be reversed by “re-opening” those windows of plasticity (86). Hyman and Nestler (87) have underscored the importance of shifting the brain into an “adaptive state” to necessitate the antidepressant response. Their theory of “initiation and adaption” is exemplified by psychotropic drugs wherein primary molecular targets that initiate alterations in brain function activate homeostatic mechanisms that return the system to an adaptive and treatment responsive state (87). Plasticity and cellular resilience are thus necessary for the efficacy of antidepressants and mood stabilizing treatments. McGorry and colleagues (6, 88) and others (89) have demonstrated this concept with pre-psychotic interventions, and repeatedly emphasized the need to take advantage of the “windows of opportunity” present within the prodromal stages of psychiatric disease (6, 88, 89). During this stage, the course of the disease remains theoretically plastic and amenable to intervention (90). Previous literature indicates that once risk or prodromal symptoms of mood disorders are identified, there is some (91), but not unequivocal (92) evidence that early intervention in adolescents can significantly reduce mood-related symptoms and incidence of fully diagnosable psychiatric disorders such as depression (93–95). Neuroprotective pharmacotherapies together with appropriate psychotherapy may reduce the risk of neuropsychiatric disease progression in young people which, together with allostatic load reducing behavioral interventions, may significantly slow the trajectory of the disease course into adulthood (6, 36, 96). Such interventions may include reducing lifestyle mediators of allostatic load (19, 97).

### Cognitive and behavioral interventions to buffer stress and build resilience

Individuals provided with effective social and emotional support to help cope with stressors that are adverse and potentially taxing will be much better placed to limit associated biological costs and maintain allostasis (98). The absence of emotional or social support and the implementation of maladaptive coping strategies can enhance the toxic effects of stress and contribute to allostatic overload (98). Exposure to regular and controllable stressors over the course of childhood and adolescence is essential for the development of effective coping strategies. Through such exposure, an individual can develop a repertoire of these coping strategies. Mathew and Nanoo (99) found that adaptive coping strategies (e.g., employing self-control, accepting responsibilities, problem solving, seeking social support, or positive re-appraisal) are protective for suicide risk in adolescents. Conversely, maladaptive coping strategies, such as confrontation, distancing, and escape-avoidance were reported to be significant risk factors associated with adolescent suicide attempts (99). These findings provide evidence to support the notion that coping strategies can act as protective factors against both the development and progression of mood disorders. Importantly, educating children and adolescents in protective coping skills may be a promising intervention that could be implemented as early as elementary school. In recent years, patterns of threat perception such as optimism have attracted much attention in relation to later mood, coping, and immune change in response to stress (100, 101). Moreover, it has been found to be protective against the development of depressive symptoms in later life (102). Its potential role in buffering against the negative emotional consequence of adverse events has led to a view of optimism as an index of resilience (103). Optimists may also choose lifestyles that promote physical as well as mental health, thereby reducing other aspects of allostatic load.

Healthy lifestyle, similar to optimism, provides a solid foundation for adaptation, and increases available resources for buffering the neurodegenerative effects of stress. Specifically, previous literature highlights the importance of healthy diet, adequate sleep, avoidance of smoking, and sufficient exercise (104). A population-based study reported higher emotional well-being among physically active youths, independent of social class and health status (105). Across a 2-year period, Motl and colleagues (106) found changes in physical activity were inversely related to a change in depressive symptoms. Levels of physical activity in childhood can modulate the risk of adult depression (107). Exercise modulates many of the core biomarkers of neuroprogression, including inflammation, oxidative stress, and neurotrophins (108). Poor eating habits and sleep have been linked to the manifestation of toxic stress and unhealthy growth in pediatrics by disrupting the architecture of the plastic, adaptive brain (109). There is now extensive evidence that poor diet quality is a risk for adolescent depression (110), and new data suggests that maternal diet influences the mental health of offspring (111). Similarly, smoking increases the risk of mood and anxiety disorders, and appears to influence similar biological pathways (112, 113). Parents and care givers of younger children need to be informed of the potential impact that a healthy lifestyle can have in mitigating mood-related symptoms and problematic behaviors. Low-risk interventions such as those aforementioned are critical for enhancing both psychological and biological resilience to stress. When such perspectives and lifestyle health behaviors are consolidated early in childhood and adolescence, the cumulative effect may be meaningful (103).

## Conclusion

Early intervention offers the possibility of altering the trajectory of mood disorder pathology. In so doing, we may curtail the progressive nature of the illness, both through neuroprotection and maintenance of peripheral health. Prevention and intervention treatments should go beyond stabilizing mood to include various and complementary strategies for reducing allostatic load, perhaps through psychoeducation and lifestyle-related interventions, including effective stress management. The combination of these techniques with specific pharmacotherapies may significantly improve functional outcomes by both reducing cellular insults and enhancing resilience. In so doing, this optimizes the capacity for maintenance of synaptic integrity and cellular resilience, which must be aggressively targeted as a therapeutic strategy during the prodromal stage of mood disorder pathology (90). This neuroprotective approach not only slows neuroprogression associated with the disease, but lays a foundation for more treatment-responsive outcomes during later stages.

## Author Contributions

Adam J. Walker, Yesul Kim, J. Blair Price, Rajas P. Kale, Jane A. McGillivray, Michael Berk, and Susannah J. Tye each made contributions to the writing of this manuscript.

## Conflict of Interest Statement

Michael Berk has received grant/research support from the NIH, Cooperative Research Centre, Simons Autism Foundation, Cancer Council of Victoria, Stanley Medical Research Foundation, MBF, NHMRC, Beyond Blue, Rotary Health, Geelong Medical Research Foundation, Bristol Myers Squibb, Eli Lilly, GlaxoSmithKline, Meat and Livestock Board, Organon, Novartis, Mayne Pharma, Servier and Woolworths, has been a speaker for Astra Zeneca, Bristol Myers Squibb, Eli Lilly, GlaxoSmithKline, Janssen Cilag, Lundbeck, Merck, Pfizer, Sanofi Synthelabo, Servier, Solvay, and Wyeth, and served as a consultant to Astra Zeneca, Bristol Myers Squibb, Eli Lilly, GlaxoSmithKline, Janssen Cilag, Lundbeck, Merck, and Servier. The other authors have no conflicts to report.
